# Attenuating effect of pretreatment with Yiqifumai on lipopolysaccharide-induced intestine injury and survival rate in rat

**DOI:** 10.1186/1476-9255-8-10

**Published:** 2011-05-02

**Authors:** Qing Yuan, Jing Wang, Qiu-Hong Fang, Yu-Ying Liu, Jing-Yu Fan, Shu-Wen Zhang, Ying-Min Ma

**Affiliations:** 1 Department of Respiratory Medicine, Beijing Shijitan Hospital Beijing, China; 2 Tasly Microcirculation Research Center, Peking University Health Science Center, Beijing, China; 3 Department of Infectious Disease, Beijing Friendship Hospital Affiliated to Capital University of Medical Science, Beijing, China, Beijing, China

**Keywords:** intestinal injury, ICAM-1, MPO, inflammatory markers, lipopolysaccharide, Chinese medicine

## Abstract

**Background:**

Yiqifumai is a traditional Chinese medicine compound preparation used for treatment of microcirculatory disturbance-related diseases in China. We have previous reported that pretreatment with Yiqifumai could improve the lipopolysaccharide (LPS) -induced microcirculatory disturbance in rat mesentery. The present study intended to investigate the effect of pretreatment with Yiqifumai on intestine injury and survival rate of the rats subjected to LPS challenge.

**Methods:**

Male Wistar rats were continuously infused with LPS (5 mg kg^-1 ^body weight h^-1^) via the left jugular vein for 90 min. In some rats, Yiqifumai 80 (mg/kg) was administrated through the left jugular vein 10 min before LPS infusion. The mean arterial pressure (MAP), heart rate (HR), rectal temperature (RT), respiratory rate (RR) and survival rate were measured at 24 h, 48 h and 72 h after LPS infusion. At 72 h after exposure to LPS, the intestine morphology was observed under a stereomicroscope and the immunohistochemistry staining of intestine was conducted to evaluate the expression of intercellular adhesion molecule 1 (ICAM-1) and the number of myeloperoxidase (MPO) positive cells in tissue. After observation of intestine microcirculation, blood was collected from the abdominal aorta of each animal to analyze the level of inflammatory markers in plasma, including TNF-α and MCP-1.

**Results:**

Compared to the control, LPS infusion significantly decreased MAP and the survival rate and increased the HR, RT and RR, as well as elicited leukocyte infiltration, intestine hemorrhage, enhanced expression of ICAM-1 and raised level of inflammatory markers. All of indicators, except for the RT, were significantly attenuated by Yiqifumai, in contrast to the LPS group.

**Conclusions:**

The results demonstrated the potential of pretreatment with Yiqifumai to ameliorate rat intestine injury, inflammatory response to LPS and the decrease in survival rate caused by LPS challenge.

## Background

Despite decades of efforts and significant advances in antimicrobial therapy and overall medical care, sepsis is still among the leading causes of death in noncardiac intensive care units, the mortality reaching to 35% in US [1] and up to 50% in China for the severe sepsis [2]. Therefore, management of sepsis remains a challenge for clinician.

Sepsis is associated with deleterious functional and structural changes in various organs, including gastrointestinal tract. Manifestations of gram-negative sepsis and septic shock are triggered by lipopolysaccharide (LPS), a component of the outer cell wall of gram-negative bacteria [3], which has long been recognized to give rise to a variety of inflammatory response as well as microcirculatory disturbance. Sepsis may relate to the impairment of microcirculation that compromises local oxygen delivery [4]. Thus, strategies potentially able to attenuate microcirculation disorders during sepsis may improve outcome.

Yiqifumai is a newly developed injection of traditional Chinese medicine that was approved in 2007 by the China State Food and Drug Administration for treatment of microcirculatory disturbance-related diseases, such as coronary heart disease, cerebrovascular disease extensively, in China. Yiqifumai consists of the water-soluble compounds of Radix Ginseng (RG), Raidix Ophiopogonis (RO) and Fructus Schisandra (FS). Available evidence revealed that components of Yiqifumai are effective for prevention and recovery of shock, ischemic and oxidative damage in the brain during heatstroke [5], able to protect against heat stroke-induced arterial hypotension and cerebral ischemia by inhibition of inducible nitric oxide synthase (iNOS)-dependent nitric oxide (NO) overproduction in the brain and excessive accumulation of inflammatory cytokines in the peripheral blood [6]. Our previous study demonstrated that administration of Yiqifumai could inhibit the leukocyte adhesion to venular wall, the degranulation of mast cell in vivo, the hydrogen peroxide (H_2_O_2_) release and the expression of adhesion molecule CD11b/CD18 in neutrophils stimulated by LPS [7]. It would be interesting to know whether or not the capacity of Yiqifumai to attenuate microcirculatory disturbance induced by intestine injury may ultimately benefit the outcome of the affected animals. The present study demonstrated the potential of Yiqifumai to improve the insults on rat imposed by infusion of LPS, including the disturbance in vital signs, the injury on intestine and the decrease in survival rate, providing further and more relevant evidence for the clinic use of Yiqifumai.

## Methods

### Reagents

LPS (Escherichia Coli serotype O55:B5) was obtained from Molecular Probes, Ltd (Eugene, OR, USA). Haemolysin were purchased from BD Biosciences Immunocytometer Systems (San Jose, CA, USA). MCP-1 and TNF-α ELISA kits were purchased from R&D Systems (Minneapolis, MN), Assay Designs (Ann Arbor, MI) or eBioscience (San Diego, CA).

Yiqifumai was obtained from Tasly Pharmaceutical Co. ltd (Tianjin, China). The lot number of the drug used in this experiment was 20070702 with a package of 0.65 g per ampoule, 1 g of Yiqifumai containing 6.3 mg of gensenoside (ingredient of RG), 253.6 mg of polysaccharides (ingredient of raidix ophiopogonis), and 0.2 mg of schizandrin (ingredient of FS) [7]. No any steroid was included in the content of Yiqifumai. The compound was dissolved in saline to a concentration of 80 mg/ml before use.

### Animals

Male Wistar rats weighing 200~250 g were obtained from the Animal Center of Peking University Health Science Center. The rats were fed a standard laboratory chow diet and maintained at 24 ± 1°C, relative humidity 50% ± 1% with a 12-h-12-h light-dark cycle. The animals were fasted for 12 h before the experiment, allowing free access to water. All animals were handled according to the guidelines of the Peking University Animal Research Committee.

### Administration of LPS and Yiqifumai

In the control group (control), 1 mL saline was injected via the left jugular vein within 1 min. Saline (6 mL kg^-1 ^body weight h^-1^) was continuously infused 10 min later through the left femoral vein for 90 min.

In the LPS group (LPS), 1 mL saline was injected via the left jugular vein within 1 min, and LPS in saline was continuously infused 10 min later through the left femoral vein for 90 min at a dose of 5 mg kg^-1 ^body weight h^-1^.

In the Yiqifumai plus LPS group (Yiqifumai+LPS), Yiqifumai saline solution (80 mg kg ^-1 ^body weight) was injected via the left jugular vein within 1 min, and LPS in saline was continuously infused 10 min later through the left femoral vein for 90 min at the same dose as in LPS group.

A total of 54 rats were included and randomly distributed into Control, LPS and Yiqifumai+LPS groups, 18 animals for each. The mean arterial pressure (MAP), heart rate (HR), rectal temperature (RT), respiratory rate (RR) and survival rate were estimated every 24 h. The examinations on intestine hemorrhage, histology and immunohistochemistry were performed at 72 h after LPS infusion on the survival animals in each group.

### Physiological parameters and survival rate

The physiological parameters were monitored at 24 h, 48 h and 72 h after the LPS infusion. Both MAP and HR were monitored with an intellectual non-invasive hemomanometer (BP-98A, Softron, China). RT was monitored by a thermocouple (ME04008, Bowdoinham, USA), and RR was counted and recorded simultaneously. At each time point, the number of survival animals was registered and the survival rate was calculated.

### Detection of hemorrhage in intestine

Surgical procedure was performed as previously described [7]. The rats were anesthetized with urethane (1.25 mg kg^-1 ^body weight) by intramuscular injection. The jugular vein and femoral vein were cannulated for injection with various reagents. For the rats that survived 72 h of LPS infusion, the abdomen was opened via a midline incision of 20-30 mm in length and ileocecal portion of the intestine was gently exteriorized and mounted on a transparent plastic stage. The intestine was kept warm and moist by continuous superfusion with saline solution at 37°C. The intestine were observed under a stereomicroscope (SZ-CTV, Olympus, Japan). Camera (Jk-TU53H, Toshiba, Japan) mounted on the microscope transmitted the images onto a color monitor (J2118A, TCL, China), and the images were recorded with a DVD (DVR-R25, Malata, China). For each animal, five intestine areas were randomly selected for observation, each of which involved five arteries and five veins that were parallel to each other and without bend [8-12]. The number of hemorrhagic spots was scored and presented as the mean of three measurements at an area

### Histologic and immunohistochemical examination of the intestine

At 72 h after exposure to LPS, 2 cm ileocecal portion of intestine was isolated and fixed with 10% buffered formalin. The samples were further processed as routine, and mounted sections were stained with hematoxylin and eosin for light microscopy. For immunohistochemical assessment, the sections were incubated with either mouse anti-intercellular adhesion molecule 1 (ICAM-1) (BD Biosciences Pharmingen, 1:400) or rabbit anti-myeloperoxidase (MPO) (NeoMarkers, Fremont, CA,1:300) overnight at 4°C, followed by incubation with biotinylated donkey anti-rabbit or donkey anti-mouse IgG (1:200) for 30 min. Positive staining was revealed by diaminobenzidine, according to the manufacture's instruction of the ABC kit (Santa Cruz Inc.). The relative integrated optical density of immunohistochemistry staining of ICAM-1 in vascular endothelium was calculated with Imaging-Pro Plus 6.0[13]. Each lesion was assessed by estimating the area of the objects and the medium pixel intensity per object, as the integrated optical density (IOD). The image background was set at white (grayscale level = 255) for IOD calculations. The number of MPO positive cells was counted within a field of view under the microscope with a 20 × objective lens and 5 fields were selected randomly in each section with the same software.

### Detection of inflammatory markers in plasma

After observation of intestine microcirculation, blood was collected from the abdominal aorta of each animal and anticoagulated with heparin (20 unit/ml blood). The plasma was isolated by centrifugation. Fifty microliters of plasma or standard was incubated with 50 μl capture beads for 1 h at room temperature, and then mixed with 50 μl phycoerythrobilin (PE)-labeled TNF-α and MCP-1 detection antibodies and incubated for 2 h at room temperature to form a sandwich complex. Following incubations, 1 ml of washing buffer (BD, Biosciences Pharmingen, USA) was added to each tube, and the mean fluorescence intensity was detected using flow cytometry (FACSCalibur, B.D. Co, USA). The data were analyzed with BD Cytometric Bead Array analysis software [10].

### Statistical analysis

All values were presented as mean ± SE. Chi-square test was applied to test for the significance of the difference in the survival rate of various groups. For the remaining parameters, the means of different groups were compared by ANOVA and F-test. A value of P < 0.05 was designed as significant.

## Results

### Survival rate and physiological parameters

**Figure 1:**
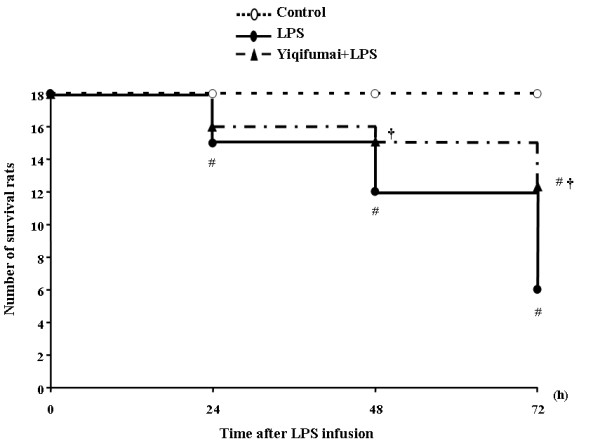
**The effect of Yiqifumai on the number of the survival rats**. Control: Control group; LPS: LPS group. Yiqifumai+LPS: Yiqifumai plus LPS group. #P < 0.05 vs. Control group, †P < 0.05 vs. LPS group.

Figure 1 shows the number of the survival rats at different time points in the 3 groups. All rats in the control group survived the 72 h observation, while the number of survival animals in LPS group decreased linearly with time and only 6 rats left at 72 h, representing a survival rate of 33%. Pretreatment with Yiqifumai significantly restored the survival rate of animals reduced by LPS exposure, reaching to 89% at 48 h, and 67% at 72 h.

**Figure 2:**
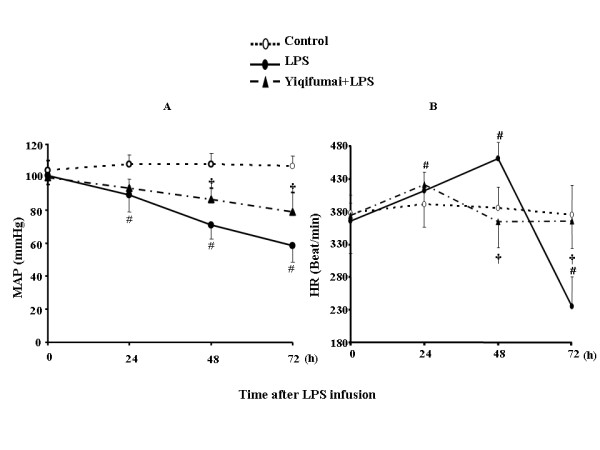
**The effect of Yiqifumai on rat mean arterial pressure (A) and heart rate (B)**. Control: Control group; LPS: LPS group. Yiqifumai+LPS: Yiqifumai plus LPS group. Data were expressed as mean ± SE. The number of animals examined at 0, 24, 48 and 72 h in each group were 18, 18, 18 and 18 for Control group, 18, 15, 12 and 6 for LPS group and 18, 16, 15 and 12 for Yiqifumai+LPS group, respectively. #P < 0.05 vs. Control group, †P < 0.05 vs. LPS group.

The time course of changes in MAP in different groups is present Figure 2(A). Obviously, the value of MAP in control group maintained almost unchanged over the period of 72 h observation. By contrast, MAP in LPS group significantly deceased at 24 h, and further deteriorated until 72 h. Pretreatment with Yiqifumai significantly attenuated MAP reduced by LPS infusion.

The time course of changes in HR under various conditions is summarized in Figure 2(B) Similarly to MAP, the value of HR in the control group kept nearly consistent during the whole period of observation. LPS infusion elicited an increase in HR at 24 h and 48 h and an impressive decrease at 72 h. Pretreatment with Yiqifumai remarkably attenuated the LPS-induced HR fluctuation.

**Figure 3:**
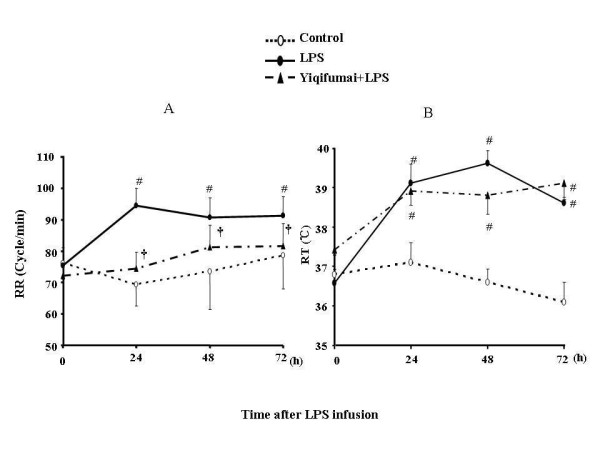
**Effects of Yiqifumai on rat respiratory rate (A) and rectal temperature (B)**. Control: Control group; LPS: LPS group; Yiqifumai+LPS: Yiqifumai plus LPS group. Data were expressed as mean ± SE. The number of animals examined at 0, 24, 48 and 72 h in each group were 18, 18, 18 and 18 for Control group, 18, 15, 12 and 6 for LPS group and 18, 16, 15 and 12 for Yiqifumai+LPS group, respectively. # P < 0.05 vs. Control group, †P < 0.05 vs. LPS group.

Left panel of Figure 3 illustrates the changes of RR with time in the three groups. The result demonstrated that in control group, RR had no significant change throughout the observation compared with baseline. In the LPS infusion group, the RR increased from 24 h after LPS infusion. Pretreatment with Yiqifumai significantly attenuated the increase in RR by LPS.

The time course of changes in RT is presented in Figure 3, right panel. Likewise, RT had no significant changes in control group throughout the period of observation compared with baseline. In the LPS infusion group, RT increased significantly starting from 24 h after LPS infusion. Pretreatment with Yiqifumai had no significant influence on the increase in RT by LPS.

### Intestine hemorrhage

**Figure 4:**
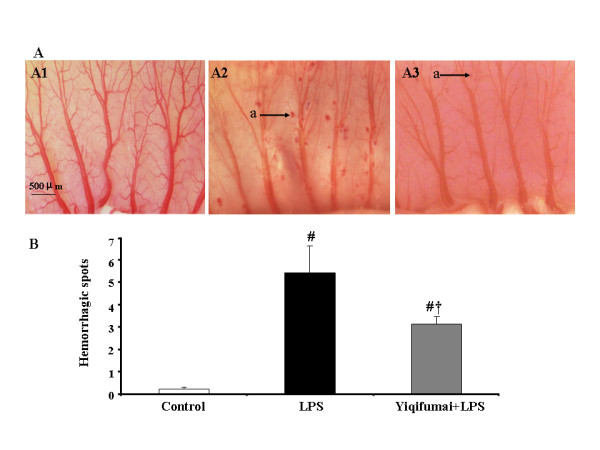
**The attenuating effect of Yiqifumai on LPS-induced rat intestine hemorrhage**. A, representative images of the intestines from different groups at 72 h after LPS infusion. In control group few hemorrhagic spots were observed (A1), numerous hemorrhagic spots in intestine were observed at 72 h after LPS infusion (A2), which was attenuated apparently by pretreatment with Yiqifumai (A3). B, quantitative evaluation of the effect of pretreatment with Yiqifumai on the hemorrhage in rat intestine elicited by LPS challenge. Data were expressed as mean ± SE. The number of animals examined was 18 for Control group, 6 for LPS group and 12 for Yiqifumai+LPS group. Arrow indicates hemorrhagic spot (a). # P < 0.05 vs. Control group, †P < 0.05 vs. LPS group.

Representative images of intestine are showed in upper panel of Figure 4, which were taken by a stereomicroscope at 72 h after LPS infusion. In control group, few, if any, hemorrhagic spots were observed (A1). However, LPS infusion for 72 h gave rise to numerous hemorrhagic spots in intestine (A2), which was attenuated apparently by pretreatment with Yiqifumai (A3). The lower panel of Figure 4 is a quantitative evaluation of the effect of pretreatment with Yiqifumai on the hemorrhage in rat intestine elicited by LPS challenge.

### Histologic study of the intestine

**Figure 5:**
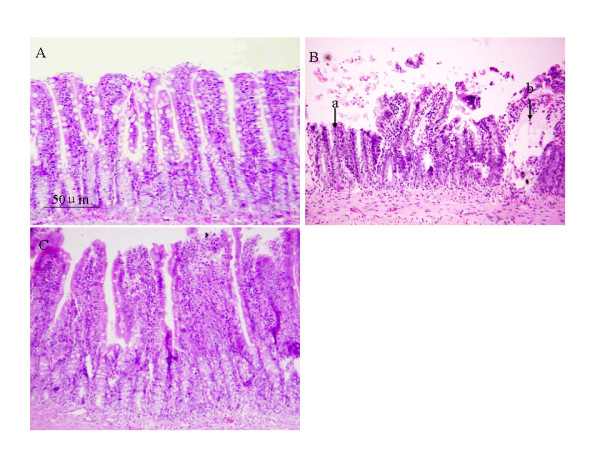
**The effect of pretreatment with Yiqifumai on the histology of rat intestine**. The morphology of intestine was examined 72 h after LPS infusion. In Control group (A), intestine mucosa was lined by densely and regularly packed villi with continuous basement membrane. No hemorrhage or swollen villus was observed. In LPS group (B), evident villus denudation (a) and swelling (b) was noted. Pretreatment with Yiqifumai significantly ameliorated the LPS-induced intestine damage (C), although some villus denudations remained.

Histologic examination on the intestine was carried out 72 h after LPS infusion, and the representative images from different groups are presented in Figure 5. In comparison with Control group (A), LPS infusion for 72 h resulted in apparent damages on the intestine mucosa, including denudation of villi with exposed dilated capillaries, lamina propria disintegration, ulceration, and hemorrhage (B). Pretreatment with Yiqifumai extenuated all the alterations induced by LPS (C).

### Immunohistochemical assessment on intestine

**Figure 6:**
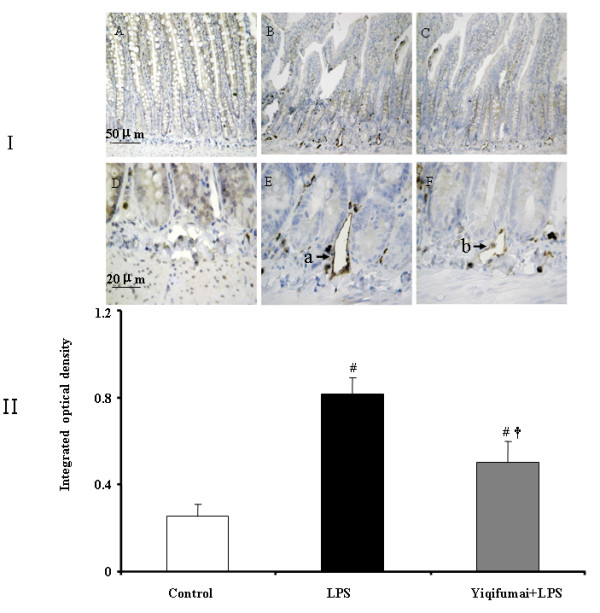
**The attenuating effect of Yiqifumai on LPS-induced expression of ICAM-1 on microvascular endothelium of intestine**. Ι, representative images of the intestines from different groups at 72 h after LPS infusion. A and D: Control group, the expression of ICAM-1 was hardly observed; B and E: LPS group, the expression of ICAM-1 was apparent (a); C and F: Yiqifumai+LPS group, the expression of ICAM-1 diminished significantly (b). ΙΙ, quantitative evaluation of the effect of pretreatment with Yiqifumai on expression of ICAM-1 on microvascular endothelium of intestine. Data were expressed as mean ± SE. The number of animals examined was 18 for Control group,6 for LPS group and 12 for Yiqifumai + LPS group. #P < 0.05 vs. Control group, †P < 0.05 vs. LPS group.

To assess the expression of ICAM-1 on microvascular endothelium of intestine, immunohistochemical staining was performed 72 h after LPS infusion. As noticed from Figure 6, rare expression of ICAM-1 was detected on microvascular endothelium of intestine in Control group (A and D). In contrast, the immunochemical staining for the expression of ICAM-1 was prominent in LPS group (B and E), which was attenuated significantly by pretreatment with Yiqifumai (C and F). This result was confirmed by a quantitative evaluation, as showed in Figure 6.

**Figure 7:**
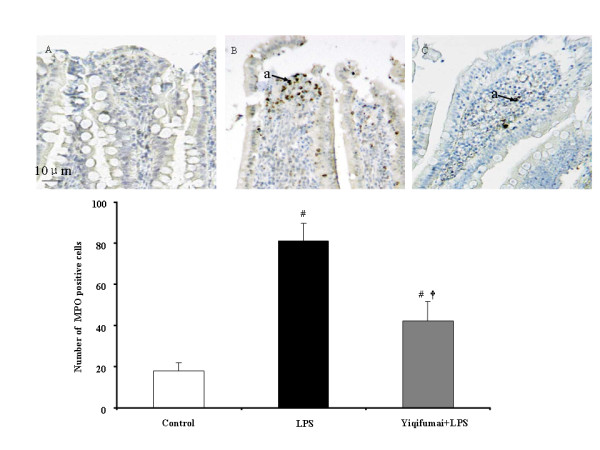
**The improving effect of Yiqifumai on the number of MPO-positive cells in intestine**. I, representative images for the effect of pretreatment with Yiqifumai on the number of MPO-positive cells in intestine. Few MPO-positive cells were observed in rat intestine in Control group (A). LPS infusion evoked numerous MPO-positive cells (B). The number of MPO-positive cells was reduced apparently by pretreatment with Yiqifumai (C). Arrow indicates MPO-positive cell (a). II, quantitative evaluation of the effect of pretreatment with Yiqifumai on the number of MPO-positive cells in intestine. Control: Control group; LPS: LPS group. Yiqifumai+LPS: Yiqifumai plus LPS group. Data were expressed as mean ± SE. The number of animals examined was 18 for Control group, 6 for LPS group and 12 for Yiqifumai + LPS group. #P < 0.05 vs. Control group, †P < 0.05 vs. LPS group.

As an indicative enzyme of neutrophil granulocytes, MPO was revealed by immunohistochemical staining 72 h after LPS infusion on intestines to evaluate the leukocyte infiltration. The representative images and the quantifications of the MPO-positive cells in the three groups are presented in Figure 7. The MPO-positive cells was hardly detectable in Control group(A) but significantly increased by LPS, indicative of leukocyte infiltration in this situation(B). Noticeably, the LPS elicited increase in MPO-positive cells was significantly inhibited by administration of Yiqifumai(C).

### Determination of concentration of inflammatory markers in plasma

**Figure 8:**
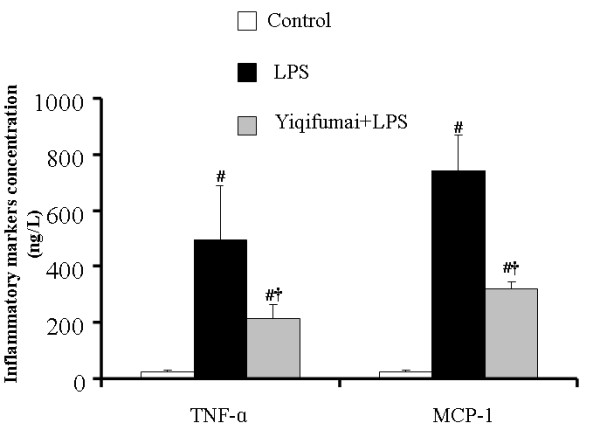
**The effect of Yiqifumai on the inflammatory markers concentration in plasma**. Control: Control group; LPS: LPS group. Yiqifumai+LPS: Yiqifumai plus LPS group. #P < 0.05 vs. Control group, †P < 0.05 vs. LPS group. The number of animals examined was 18 for Control group, 6 for LPS group and 12 for Yiqifumai + LPS group.

The concentrations of the cytokines TNF-α and MCP-1 in plasma are presented in Figure 8. In the control group, the concentrations of TNF-α and MCP-1 were 24.51 ± 5.57 and 22.72 ± 8.67 ng/l, respectively. The concentrations of the two cytokines were enhanced dramatically by LPS stimulation to 493.97 ± 192.29 and 741.53 ± 126.86 ng/l, respectively. Treatment with Yiqifumai inhibited the LPS-induced production of TNF-α and MCP-1 markedly.

## Discussion

The present study demonstrated that LPS continuous infusion caused a range of disturbances in the vital signs of rats, including the decrease in MAP and the increase in HR, RT and RR, as well as raised the level of inflammatory markers in plasma and reduced the survival rate of the animals. Morphological examination showed a significant increase in leukocyte infiltration, hemorrhage and expression of ICAM-1 in intestine in response to LPS infusion. Interestingly, all the LPS elicited alterations, except for RT, were ameliorated attenuated significantly by pretreatment with Yiqifumai.

LPS is responsible for the initiation of the septic cascade in Gram-negative bacterial infections, which involves upregulation of intercellular adhesion molecules 1 (ICAM-1) and production of large amount of cytokines [14,15], the two processes that are both mediated by NF-κB and interplayed each other, cumulating to the damage on the vascular endothelium [16-18]. Leukocytes bind to the vessel through interaction with ICAM-1 on the surface of the endothelium, these leukocyte-endothelial interactions promote the release of reactive oxygen species (ROS) and other mediators, which destroy bacteria on one hand, but inflict damage on the endothelium and cause exaggerated microvascular dysfunction on the other hand [19]. The endothelial permeability thus increases due to the structure damage of the endothelial cells as well as to the enzymatic cleavage of adherent junction proteins [20], which eventually results in the transmigration of leukocytes across the endothelial lining into the surrounding tissues and the loss of fluid into extravascular space leading to hypotension and life-threatening edema in multiple organs [21]. It is known that the MPO activity was markedly relevant to tissue neutrophil accumulation [22,23]. In this study, the intestine tissue MPO activity was assayed to assess the numbers of neutrophils recruited to the intestine tissue. The sepsis-associated organ injury is further complicated by the implication of overproduction of reactive oxygen species [24,25]. Cytokines such as TNF-α and MCP-1, which are believed to be pro-inflammatory factors, are produced by activated monocyte/macrophages, and acts mainly to attract neutrophils and monocytes [26]. These chemokines are all associated with the influx, accumulation and activation of highly destructive cells involved in local inflammatory processes. As Wang has reported Shengmai San, which has similar ingredients with Yiqifumai, significantly reduced excessive accumulation of inflammatory cytokines in the peripheral blood[5]. We have acquired the similar outcomes in this study, Yiqifumai alleviated remarkably the overproduction of inflammatory markers(TNF-α and MCP-1). Owing to the complexity of the events in the course of sepsis, it is unlikely that a single therapeutic agent, especially those that target the points downstream the network, such as inhibition of inducible nitric oxide synthase (iNOS)-dependent nitric oxide (NO) overproduction, may overcome all the complications. It would be desirable to have a regime that not only acts at the crucial initial steps of sepsis but also has multi-targeting potential. We have previously reported that pretreatment with high-dose Yiqifumai (80 mg kg^-1^), compared to low (15 mg kg^-1^) and medium (30 mg kg^-1^) dose group, significantly attenuates LPS-induced microcirculatory disturbance in rat mesentery, including reduction in the number of adherent leukocytes, the intensity of DHR fluorescence, degradation of mast cell, albumin leakage, and the expression of CD11b/CD18, suggesting that Yiqifumai is a promising regime for treatment of LPS-evoked sepsis thanks to its multiple targeting potential for the initial steps of the process [7]. The results of the current study demonstrated this speculation, showing that pretreatment with Yiqifumai significantly ameliorates the outcome of the rats exposed to LPS, exhibiting as the improvement of the vital signs, attenuation of the intestine injury and the increase in survival rate.

**Figure 9:**
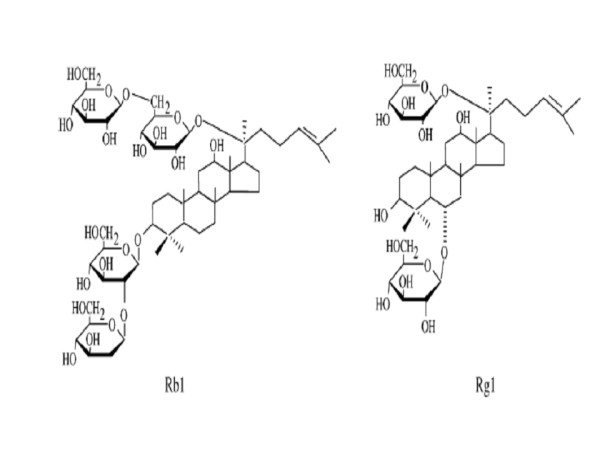
**Structures of ginsenoside Rb1 (Rb1), ginsenoside Rg1 (Rg1), major active components of RG**.

The beneficial effects found for Yiqifumai in the present study may be attributed at least in part to RG, one of the ingredients of Yiqifumai. We have previous reported that Ginsenoside Rb1 (Rb1) and Ginsenoside Rg1 (Rg1), whose chemical structure were presented in Figure 9, the major active components of RG, could both inhibit the leukocyte adhesion to venular wall and the degranulation of mast cell in vivo, and Rg1 may attenuate the hydrogen peroxide (H_2_O_2_) release and Rb1 attenuate the expression of adhesion molecule CD11b/CD18 in neutrophils stimulated by LPS [10]. We assume that it is these actions, together with the finding in the current study that Yiqifumai inhibits the expression of ICAM-1, that attenuate the recruitment of leukocytes and endothelium dysfunction, reduce the leukocyte infiltration and edema in the surrounding tissues and subsequent organ injury. The exact role of other two ingredients of Yiqifumai, RO and FS, requires further identification, although the advantage of using three ingredients over RG alone is evident.

## Conclusions

In summary, the study demonstrated that pretreatment with Yiqifumai significantly improve the outcome of the rats that were subject to LPS challenge, and this effect may be associated with the actions that Yiqifumai exerted on the pivotal initial points of the sepsis process. This result provides further support for the use of Yiqifumai as an adjuvant therapy for sepsis in clinic.

## Competing interests

The authors declare that they have no competing interests.

## Authors' contributions

QY carried out the intestine microcirculatory, survival rate, physiological parameters observation and drafted the manuscript, JW participated in the histologic study and cytokines detection, Q-H F carried out the immunohistochemical assessment. Y-Y L and J-Y Fan participated in the design of the study and performed the statistical analysis. S-W Z and Y-M M conceived of the study, and participated in its design and coordination and helped to draft the manuscript. All authors read and approved the final manuscript.
